# Activated Hepatic Stellate Cells in Hepatocellular Carcinoma: Their Role as a Potential Target for Future Therapies

**DOI:** 10.3390/ijms232315292

**Published:** 2022-12-04

**Authors:** Esraa Ali, Andriy Trailin, Filip Ambrozkiewicz, Václav Liška, Kari Hemminki

**Affiliations:** 1 Laboratory of Translational Cancer Genomics, Biomedical Center, Faculty of Medicine in Pilsen, Charles University, Alej Svobody 1665/76, 32300 Pilsen, Czech Republic; 2 Laboratory of Cancer Treatment and Tissue Regeneration, Biomedical Center, Faculty of Medicine in Pilsen, Charles University, Alej Svobody 1665/76, 32300 Pilsen, Czech Republic; 3 Department of Surgery University Hospital and Faculty of Medicine in Pilsen, Charles University, Alej Svobody 80, 32300 Pilsen, Czech Republic; 4 Department of Cancer Epidemiology, German Cancer Research Center, Im Neuenheimer Feld 280, 69120 Heidelberg, Germany

**Keywords:** hepatocellular carcinoma, hepatic stellate cells, fibrosis regression, therapeutic studies

## Abstract

Hepatocellular carcinoma (HCC) is a global healthcare challenge, which affects more than 815,000 new cases every year. Activated hepatic stellate cells (aHSCs) remain the principal cells that drive HCC onset and growth. aHSCs suppress the anti-tumor immune response through interaction with different immune cells. They also increase the deposition of the extracellular matrix proteins, challenging the reversion of fibrosis and increasing HCC growth and metastasis. Therapy for HCC was reported to activate HSCs, which could explain the low efficacy of current treatments. Conversely, recent studies aimed at the deactivation of HSCs show that they have been able to inhibit HCC growth. In this review article, we discuss the role of aHSCs in HCC pathophysiology and therapy. Finally, we provide suggestions for the experimental implementation of HSCs in HCC therapies.

## 1. Introduction

Liver cancer is a global health problem, with an estimated increase of 32% by 2040 [1]. Representing 90% of liver cancers, hepatocellular carcinoma (HCC) causes 700,000 deaths annually. 

**Figure 1:**
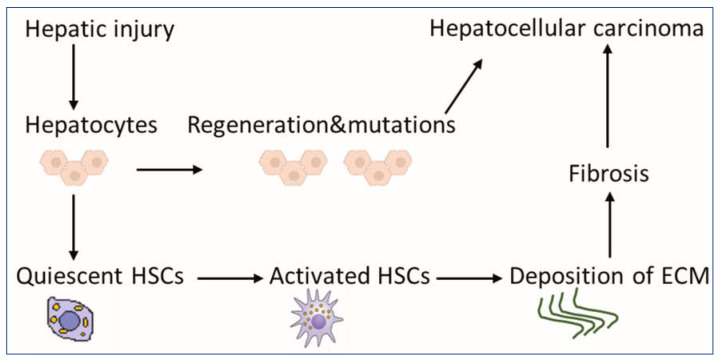
Scheme for the contribution of HSCs to liver pathology. HSCs exist in a quiescent state, containing numerous vitamin A lipid droplets. When the liver is injured, damaged hepatocytes mediate HSC activation, which could produce a large amount of ECM, leading to fibrosis as an indirect mechanism of HCC. Mutations during the regeneration of hepatocytes may lead directly to the development of HCC. Abbreviations: HSCs: hepatic stellate cells, HCC: hepatocellular carcinoma and ECM: extracellular matrix.

Although HCC pathogenesis is complex and varies depending on underlying etiology, the usual background setting for HCC is liver injury, chronic inflammation, irreversible fibrosis, and cirrhosis [2]. In fact, 80–90% of HCC develops in the fibrotic or cirrhotic liver [3]. Hepatic stellate cells (HSCs) play a key role in this sequence of events, contributing mainly to liver fibrosis and cirrhosis. They are liver-specific mesenchymal cells, which are located in the perisinusoidal space in contact with different cell types [4]. In a healthy liver, HSCs exist in a quiescent non-proliferative state as an important source of paracrine, autocrine, and chemoattractant factors to maintain hepatic homeostasis [5]. Quiescent HSCs are very sensitive to extracellular pro-fibrotic signals [6] and contain numerous vitamin A lipid droplets, which are essential for the proper function of the immune system [7]. When toxins or viruses injure the liver, damaged hepatocytes and immune cells secrete signals, which could activate HSCs into myofibroblast-like cells [8]. Activated HSCs produce an extracellular matrix (ECM) at the site of injury as a temporary protective scar to prevent further damage, initiating the first steps of fibrosis [8,9]. Long-acting agents maintain the activation of HSCs, increasing their capabilities for proliferation and migration [10]. Activated HSCs produce more ECM, leading to chronic fibrosis and cirrhosis and eventually to HCC (Figure 1) [6]. Despite significant advances in the treatment of HCC, drug-resistance is a critical obstacle [11], and the 5-year survival rate is low (5–14%) [12], but survival rates of greater than 20% have been reached in some regions [13]. Therefore, there is a potential to increase survival rates. Given the fact that available therapies can activate HSCs, synchronous targeting aHSCs may be beneficial for patients [14]. In this review, we emphasize the role of aHSCs in HCC. We clarify how aHSCs suppress the immune response in the tumor microenvironment. We provide insights into the contribution of HSCs to slow down fibrosis regression and to increase deposition of ECM proteins, which may favor HCC growth and metastasis. In addition, we focus on the ability of conventional therapies to activate HSCs, whereas studies of aHSC deactivation might be an important strategy to improve HCC treatment. Finally, we suggest how to reinforce experiments that target aHSCs.

## 2. The Role of aHSCs in HCC

### 2.1. The Suppression of the Antitumor Immune Response by aHSCs

In HCC, aHSCs receive signals from individual immune cells, and, in turn, they produce soluble mediators, acting on surrounding immune cells [15]. HSC mediators could orchestrate both innate and adaptive immunity, resulting in an immunosuppressive tumor microenvironment (Table 1) [7]. Reduction of antitumor responses was shown in immunocompetent mice after co-transplantation of HSCs and HCC cells [16]. Such cotransplantation of HSCs inhibited systemically lymphocyte infiltration, which promoted tumor cell proliferation and, therefore, HCC growth; the size of the tumors was HSC dose-dependent [16]. Previous experiments addressing HCC–HSC interactions were performed on immunodeficient mice, and, therefore, the effect of HSC on the immune system was not investigated. Using immunocompetent mice, the authors were able to define HSC-immune interactions in HCC [16].

The antigen-directed cytotoxicity of T lymphocytes (TLs) boosts the immune response against cancer [17]. Activation and proliferation of TL in tumor tissue, predominantly CD8+ and CD4+ T lymphocytes, can control HCC progression [18]. HSCs can exert their immunomodulatory activities by downregulating the number and function of CD4+ and CD8+ TLs [19]. Contrary to quiescent HSCs, aHSCs in mice and humans expressed programmed death-ligand 1 (PD-L1) to inhibit TL responses [7]. PD-L1 expressed by HSCs can induce TL apoptosis, attenuate TL infiltration, and suppress TL-mediated cytotoxicity, therefore inhibiting TL responses and enabling tumor cells to escape the host immune response [20]. In addition, HSCs may prevent the local stimulation of naive TLs [21]. In Hepa1–6 cells, activated HSCs induced the death of activated TLs and reduced the cytotoxicity of cancer-specific TLs, which resulted in the increased proliferation and migration of cancer cells [22]. More investigation of the role of aHSCs in the apoptosis of TLs in HCC patients is needed.

aHSCs also induce expansion of two suppressive immune cell populations; myeloid-derived suppressor cells (MDSCs) [14] and T helper 17 (Th17) cells, a subset of CD4+ effector T cells [23]. MDSCs play a pivotal negative role in the immune response through the inhibition of cytotoxic T cells and recruitment of regulatory T cells, which results in tumor progression [24]. HSCs induce MDSC accumulation in the tumor tissue by the stimulation of the COX2–PGE2–EP4 pathway [25]. Inhibition of this pathway in murine orthotopic HCC models downregulated MDSCs and HCC growth [25]. Immunosuppressive functions of Th17 cells may contribute to HCC progression [26]. IL-17A produced by Th17 could increase cancer cell motility via the activation of the nuclear factor-kB (NF-kB) transcript factor, increasing HCC metastasis [27]. Culturing CD4+ cells with HSCs (extracted from hepatitis B virus-related fibrotic liver tissue) increased the percentages of Th17 cells [23]. HSCs may secrete high levels of interleukin-6 as a critical initiator of Th17 expansion and tumor necrosis factor-α as a key regulator of Th17 differentiation [28]. Interestingly, previous data indicated suppression of Th17 differentiation by mouse HSCs [29]. Critical evaluation of Th17-HSCs interactions could be addressed in appropriate mouse models of HCC. 

Macrophages polarize in the liver with strong plasticity into pro-inflammatory M1 or anti-inflammatory M2 in response to local signals from the tumor microenvironment [30,31]. M1 macrophages are thought to be tumoricidal, while M2 macrophages are usually believed to promote tumorigenesis and tumor progression [32]. M2 macrophages in HCC promote the invasion and migration of tumor cells [33]. M2 macrophage-derived CCL22 was proven to enhance tumor migration through the activation of epithelial–mesenchymal transition [34]. aHSCs recruited CCL2/CCR2 pathway in HCC cell lines to stimulate M2 phenotypic transformation [35]. M2 macrophage polarization could lead to the progression of HCC [36].

Natural killer (NK) cells defend the body against tumors by engaging death-inducing receptors to stimulate cancer cell apoptosis. HCC patients with low intratumoral NK cells infiltration have shorter disease-free survival [37]. In animal models of fibrosis, transforming growth factor-β secreted by HSC could inhibit NK cell function [38]. On the other hand, NK cells could induce apoptosis of aHSCs in hepatitis C virus-infected patients [39] and mouse models of fibrosis [40]. Studies on the interaction between aHSCs and NK cells in HCC models should be investigated. Dendritic cells (DCs) can activate antitumor immunity by priming TL against cancer-progression-associated antigens. HSCs induce the expression of dendritic-cell-derived immunoglobulin receptor 2 (DIgR2), which inhibits DC-induced antigen-specific TL responses [41]. DIgR2 was shown to bind to the receptor in TLs, suppressing TL proliferation, cytokine production, and cytotoxic TL activity [41]. Co-culturing of tumor-HSCs (isolated from the tumor) to DCs induced the expression of DIgR2, in contrast to quiescent HSCs, which had no significant effect on DIgR2 expression. Considering quiescent HSCs in such studies boosts the role of activated HSCs in HCC [41].

Although the role of immune system in liver cancer is complex [42], the overall role of aHSCs in immune regulation is pro-oncogenic [16]. Exploring the interaction between different immune cells and HSCs in established HCC models could highlight the main targets to improve immune surveillance against HCC.

**Table 1:** Immunosuppressive functions of HSCs.

Mediator	Immune cell	Response	Possible Effect
PD-L1	T lymphocytes	TL apoptosis, attenuation of TL infiltration, and suppression of TL-mediated cytotoxicity [20]	HCC growth
Dendritic-cell-derived immunoglobulin receptor 2	Dendritic cells	Inhibition of DC-induced antigen-specific TL responses [41]	
COX2–PGE2–EP4	MDSCs	MDSC accumulation [25]	
Transforming growth factor-β	NK cells	Inhibition of NK cell function [38]	
Interleukin-6 and tumor necrosis factor-α	Th17	Th17 expansion and Th17 differentiation [28]	HCC metastasis
CCL2/CCR2	Macrophages	Stimulation of M2 macrophages phenotypic transformation [35]	

HSCs produce soluble mediators acting on surrounding immune cells, resulting in a negative immune response. The immune regulatory role of HSCs may lead to HCC growth and metastasis. HSCs: hepatic stellate cells, PD-L1: programmed death-ligand 1, MDSCs: myeloid-derived suppressor cells, NK: natural killer, Th17: T helper 17, HCC: hepatocellular carcinoma, and TL: T lymphocyte.

### 2.2. HSCs Upregulate the Deposition of ECM for the Development of Fibrosis and HCC 

**Figure 2:**
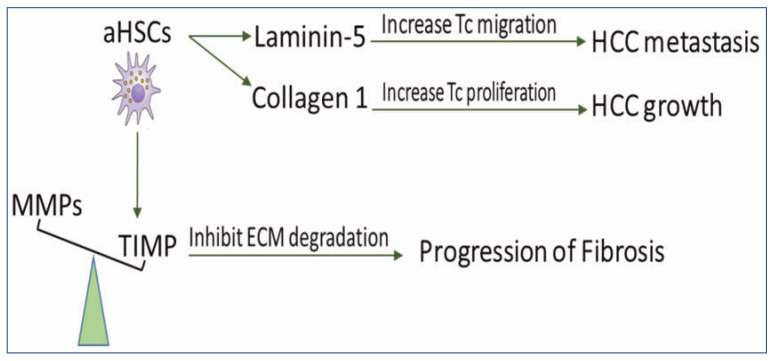
The scheme demonstrates the role of HSCs in disturbing ECM balance for the development of fibrosis and HCC. aHSCs express TIMP glycoproteins which inhibit MMP-mediated degradation of ECM and, therefore, prevent fibrosis regression. They also secrete collagen I, which increases tumor cell proliferation and produces Ln-5, which promotes tumor cell migration. MMPs: matrix metalloproteinases, TIMPs: tissue inhibitors of MMP, aHSCs: activated hepatic stellate cells, HCC: hepatocellular carcinoma, ECM: extracellular matrix, and TC: tumor cell.

Under normal conditions, the rate of ECM production in the liver equals that of its degradation, resulting in no net accumulation of the matrix. Fibrogenesis occurs when there is an imbalance between ECM production and degradation [43], resulting in the impairment of liver functions, which may eventually lead to cirrhosis and HCC [44]. Fibrosis and cirrhosis are reported clinically as reversible processes [45]. Reversibility of liver fibrosis depends mainly on the degradation of ECM [46]. aHSCs are able to increase matrix protein synthesis, which might lead to the irreversibility of fibrosis and favor progress and metastasis of HCC (Figure 2).

The reversibility of fibrosis and cirrhosis is dependent on the activity of matrix metalloproteinases (MMPs) [47]. MMPs are a group of enzymes involved in the degradation of ECM-proteins, which are blocked by tissue inhibitors of MMP (TIMPs). It has been reported that prolonged expression of TIMPs, even after withdrawal of fibrogenic factors, slows the regression of liver fibrosis [47]. In a rat model of regressed liver fibrosis, the reversibility of fibrosis was increased in parallel with a marked decrease in TIMP expression [48]. Fully activated HSCs release and upregulate expression of TIMP-1 and TIMP-2, which inactivate MMPs through proteolytic cleavage [43,45,48]. Targeting activated HSCs in vivo decreased the expression of TIMP-1 and TIMP-2 and resulted in attenuated liver fibrosis [49]. Impairment of HSCs activation in mice downregulated TIMP-1 and diminished alcohol-induced steatohepatitis [50]. Interestingly, the addition of activated MMP-2 to aHSCs in culture enhanced the apoptosis of HSCs [51].

Increased production of ECM proteins, such as collagen I and laminin-5 (Ln-5), is associated with the growth and metastasis of HCC. Collagen I promotes HCC cell proliferation by regulating the integrin β1/FAK signaling pathway [52]. HSCs produce collagen I [53], which has been associated with the increased aggressiveness of HCC [54], where silencing its expression in HSCs may treat liver fibrosis [55].

Upregulation of Ln-5 in HCC patients promotes the migration of tumor cells, which is directly related to poor prognosis and tumor metastasis [56]. HCC grows in a microenvironment enriched with Ln-5 produced by HSCs [57]. In human HCC tissues, Ln-5 was distributed mainly along aHSCs, stimulating tumor cell migration [58]. Blocking antibodies against Ln-5 in HCC cell lines in the presence of HSCs inhibited tumor metastasis [58], while the presence of HSCs or Ln-5 in HCC cell lines increased resistance to sorafenib [57].

Normalization of ECM may represent an important therapeutic strategy for HCC [59]. Analysis of HSC-secreted proteins that control components of ECM could identify possible targets for HCC treatment. 

## 3. Activation and Deactivation of HSCs as a Result of Therapy 

### 3.1. Activation of HSCs by Conventional Therapy 

Current conventional therapy can activate HSCs, which could explain its limited success in curing HCC (Table 2). Chemotherapy can cause activation of HSCs through stroma-derived factor 1 and hypoxia-inducible factor 1 α. These mechanisms often regulate, unite, or intersect with other pathways to activate HSCs [14]. Transarterial chemoembolization (TACE) is clinically recommended for patients with advanced-stage HCC. However, the long-term results of TACE in HCC might be compromised by TACE-induced hepatic hypoxia and subsequent HSC activation [60]. In HCC animal models, TACE activated HSCs and induced prominent hepatic fibrogenesis [61]. In contrast, latent HSCs countered cancer growth by increasing the cytotoxicity of chemotherapeutics such as doxorubicin [62]. Treating rat hepatoma cells with quiescent HSCs and doxorubicin enhanced the efficacy of doxorubicin and led to faster tumor cell death [62].

Sorafenib, as an anti-tumor molecular inhibitor, can impede HCC cell proliferation but can also activate HSCs through the mitogen-activated protein kinase (MAPK) signaling pathway [63]. In mice, combined delivery of sorafenib with an inhibitor for MAPK could prevent the activation of HSCs, resulting in anti-fibrotic properties [63]. Coculturing of HSC-LX2 in Huh7 cell lines induced sorafenib resistance [64]. The interaction between sorafenib and aHSC might affect the success rate of this molecular inhibitor.

The main challenge to classic radiotherapy is its side effects on the surrounding tissues [65]. Upon radiotherapy, HSCs were activated and accumulated in the patient’s liver [66]. In a hepatoma cell line, radiotherapy activated HSCs through the toll-like receptor 4 pathway and increased the potential of HCC metastasis [67]. The activation of HSCs by radiation is a key process underlying hepatic fibrosis, which could promote radioresistance and tumor recurrence [14].

Although radiofrequency ablation (RFA) is increasingly incorporated into HCC treatment, available data indicate its ability to stimulate residual tumor growth and cause tumor recurrence [68]. It was reported that RFA could activate HSC through inflammatory cytokine-mediated pathways. Elevated levels of interleukin 6 and a massive accumulation and migration of activated HSCs were recorded in mice after RFA [69].

Defining and analyzing the interconnected factors between HSC activation and conventional therapies could help to enhance the efficacy of current treatment modalities.

### 3.2. Pharmacological Approaches to Deactivate HSCs

Pharmacological trials that target activated HSCs suggest a new paradigm that “hitting one target leads to a domino effect”. This is easy to understand since targeting aHSCs will affect subsequently HSC-induced immunosuppression, drug resistance, and tumor metastasis (Table 2).

The trials are based mainly on targeting molecular pathways of HSCs activation. TGF-β signaling is considered the key pathway that drives HSC activation [70]. Targeting TGF-β emerges as an effective therapeutic option to revert the activation of HSCs and stop the progress of HCC. Galunisertib is a small-molecule selective inhibitor of TGF-β receptor type I. The combination of galunisertib and sorafenib in patients with advanced HCC showed acceptable safety and prolonged overall survival [71]. Imatinib simultaneously and rather selectively inhibits TGF-β signaling. In mice, El-Mezayen et al. targeted HSCs using imatinib–nanomedicine therapy resulting in outstanding anti-fibrotic effects with reduced cytotoxicity [72].

Another pathway for HSC activation implies the involvement of peripheral nerves. Peripheral nerves secret substance P (SP), which transmits the information via a neurokinin-1 receptor (NK-1R)—expressed on HSCs. SP activates HSCs through the SP/NK-1R signal pathway [73]. In vivo, the combination of doxorubicin with capsaicin, as a blocker to “SP-HSCs”, could effectively inhibit drug resistance and HCC metastasis [73].

PD-L1 is required for HSC activation by stabilizing TGF-β receptors [74]. Targeting HSC PD-L1 in mice suppressed HSC activation and growth of intrahepatic cholangiocarcinoma [74]. Nivolumab, a blocker of PD-L1, was applied in the treatment of advanced HCC patients who were resistant to sorafenib [75]. New therapeutic approaches that combine targeting HSCs with traditional treatment could become a gold standard to cure HCC.

## 4. Suggestions for the Application of HSCs in the Treatment of HCC

Growing evidence of the role of HSCs in HCC suggests that HSC-related therapies might revolutionize the therapy for HCC. Yet, several gaps still limit the application of HSC deactivation in the treatment of HCC. 

The first obstacle is the obscure nature of molecular mechanisms of HSC activation and deactivation [76]. In particular, quiescent HSCs shift to the activated state in a dynamic complex scenario with different subpopulations, which could orchestrate the course of HCC [77]. Filliol et al. showed how quiescent or least-activated HSCs express hepatic growth factor, which limits HCC growth, but in advanced disease stages, fully activated HSCs express collagen I, promoting tumor proliferation [77]. The signals, which modulate these HSC subpopulations, have not been investigated. In addition, during fibrosis regression, activated HSCs can undergo apoptosis or revert to an inactivate phenotype, which is distinct from quiescent HSCs [78]. We may address those challenges by analyzing gene expression signatures for quiescent, activated, and inactivated HSCs in parallel with transcriptional analysis of their cellular neighborhood during different stages of HCC. Administering HSCs to experimental animals in a different experimental setting (fibrosis, HCC) and tracking their interactions could give us a better understanding of the role of HSCs in different liver pathological conditions.

The second obstacle is that HSCs account only for 5% to 8% of total liver cells, and targeting HSCs may be blocked by the condensed nature of the perisinusoidal space [79]. Targeting HSCs with free drugs could cause severe systemic toxicity, and the benefits are restricted due to their poor solubility, short half-life, and low bioavailability [73]. The application of drug carrier systems, which deliver drugs to specific tissues [80], may solve this problem [79]. Delivering drugs to aHSCs would be expected to boost potency with decreased side effects [72]. When nanomicelles were modified to target aHSCs in vivo, they suppressed the activation of HSCs and inhibited fibrosis development safely and efficiently [81]. 

Controlled studies to define the main factors of HSC activation and the proper delivery system to target it should promote the potential of HSCs in the context of HCC therapies. The final step is to define the inclusion and exclusion criteria for such therapies. The inclusion criteria need to consider HCC staging and hepatic microenvironment, including immune cells and ECM materials.

## 5. Conclusions

HSCs play an exemplary role in the tumor regulating different cytokines and growth factors for the progress of HCC. They interact with different immune cells to suppress HCC immunosurveillance. Targeting HSC-related immune suppression would improve immune response to HCC. aHSCs disturb the balance of ECM proteins, which leads to the progression of fibrosis and HCC. Exploring how HSCs destabilize ECM could identify possible targets for HCC treatment. As current therapies activate HSCs, deactivation of HSCs has become a critical therapeutic strategy. Targeting aHSCs can be developed by studying their molecular activation mechanisms and selecting proper targeting methods.

## Figures and Tables

**Table 2:** Illustration of the effect of current treatments and new pharmacological studies on HSCs.

Approach	Effect on HSCs	Possible Result	Ref.
TACE	Activate HSCs	Induce prominent hepatic fibrogenesis	[61]
Sorafenib	Activate HSCs	Resistance to sorafenib	[64]
Sorafenib and MAPK inhibitor	Prevent HSC activation	Anti-fibrotic effect	[63]
Radiotherapy	Activate HSCs	Increase HCC metastasis	[67]
Radiofrequency ablation	Activate HSCs	Tumor recurrence	[69]
Galunisertib and sorafenib	Deactivate HSCs	Prolonged overall survival in HCC patients	[71]
Imatinib–nanomedicine	Deactivate HSCs	Outstanding anti-fibrotic effects	[72]
Doxorubicin and capsaicin	Deactivate HSCs	Inhibit drug resistance and HCC metastasis	[73]
Nivolumab	Deactivate HSCs	Treat advanced HCC	[75]

Conventional therapies are able to activate HSCs, which could improve tumor growth. In contrast, the deactivation of HSCs by new pharmacological products could inhibit HCC progress and arise as an effective therapeutic strategy. HSCs: hepatic stellate cells, TACE: transarterial chemoembolization, MAPK: mitogen-activated protein kinase, and HCC: hepatocellular carcinoma.

## Abbreviations

HCC	hepatocellular carcinoma
aHSCs	activated hepatic stellate cells
ECM	extracellular matrix
TLs	T lymphocytes
PD-L1	programmed death-ligand 1
MDSCs	myeloid-derived suppressor cells
Th17	T helper 17 cells
NF-kB	nuclear factor-kB
IL6	interleukin-6
NK	natural killer
IL-10	interleukin-10
TGFβ	transforming growth factor-β
DCs	dendritic cells
MMPs	metallo-proteinases
TIMPs	tissue inhibitors of MMP
Ln-5	laminin-5
TACE	transarterial chemoembolization
MAPK	mitogen-activated protein kinase
RFA	radiofrequency ablation
NK-1R	neurokinin-1 receptor
SP	substance P
DIgR2	dendritic-cell-derived immunoglobulin receptor 2
